# Alleviation of exhaustion-induced immunosuppression and sepsis by immune checkpoint blockers sequentially administered with antibiotics—analysis of a new mathematical model

**DOI:** 10.1186/s40635-019-0260-3

**Published:** 2019-06-11

**Authors:** Avi Gillis, Michael Beil, Karin Halevi-Tobias, Peter Vernon van Heerden, Sigal Sviri, Zvia Agur

**Affiliations:** 1 Institute for Medical BioMathematics, 10 Hate’ena St, P.O.B. 282, 60991 Bene Ataroth, Israel; 2 Medical Intensive Care Unit, Hadassah University Hospital, PO Box 12000, 9112001 Jerusalem, Israel; 3 General Intensive Care Unit, Hadassah University Hospital, PO Box 12000, 9112001 Jerusalem, Israel

**Keywords:** Simulations, Dynamic model, T lymphocyte exhaustion, Immunosuppression, Checkpoint blockers, Hyper-inflammation, Hematopoietic stem cell, Programmed cell death protein 1, PD-1, Pathogen

## Abstract

**Background:**

Sepsis-associated immune dysregulation, involving hyper-inflammation and immunosuppression, is common in intensive care patients, often leading to multiple organ dysfunction and death. The aim of this study was to identify the main driving force underlying immunosuppression in sepsis, and to suggest new therapeutic avenues for controlling this immune impairment and alleviating excessive pathogen load.

**Methods:**

We developed two minimalistic (*skeletal*) mathematical models of pathogen-associated inflammation, which focus on the dynamics of myeloid, lymphocyte, and pathogen numbers in blood. Both models rely on the assumption that the presence of the pathogen causes a bias in hematopoietic stem cell differentiation toward the myeloid developmental line. Also in one of the models, we assumed that continuous exposure to pathogens induces lymphocyte exhaustion. In addition, we also created therapy models, both by antibiotics and by immunotherapy with PD-1/PD-L1 checkpoint inhibitors. Assuming realistic parameter ranges, we simulated the pathogen-associated inflammation models in silico with or without various antibiotic and immunotherapy schedules.

**Results:**

Computer simulations of the two models show that the assumption of lymphocyte exhaustion is a prerequisite for attaining sepsis-associated immunosuppression, and that the ability of the innate and adaptive immune systems to control infections depends on the pathogen’s replication rate. Simulation results further show that combining antibiotics with immune checkpoint blockers can suffice for defeating even an aggressive pathogen within a relatively short period. This is so as long as the drugs are administered soon after diagnosis. In contrast, when applied as monotherapies, antibiotics or immune checkpoint blockers fall short of eliminating aggressive pathogens in reasonable time.

**Conclusions:**

Our results suggest that lymphocyte exhaustion crucially drives immunosuppression in sepsis, and that one can efficiently resolve both immunosuppression and pathogenesis by timely coupling of antibiotics with an immune checkpoint blocker, but not by either one of these two treatment modalities alone. Following experimental validation, our model can be adapted to explore the potential of other therapeutic options in this field.

**Electronic supplementary material:**

The online version of this article (10.1186/s40635-019-0260-3) contains supplementary material, which is available to authorized users.

## Background

Proper inflammatory responses provide broad-spectrum protection against infections, and orchestrate long-term adaptive immunity toward specific pathogens. In contrast, hyper-inflammation, resulting in major pathogenicity from overzealous immune response, can inflict severe tissue damage, multiple organ dysfunction, and ultimately death [1–4]. Immunosuppression can follow hyper-inflammation to control the potential damage to the host, by activating an anti-inflammatory response, which can occur regardless of whether or not the initial cause of the inflammation (e.g., an invading pathogen) has been resolved [5]. In their mild form, both pro- and anti-inflammatory responses are essential for the host’s protection. During a mild infection, a measured pro-inflammatory phase is sufficient for pathogen clearance, whereby a subsequent anti-inflammatory phase returns the immune system to homeostasis. However, when the infection is more severe, it can cause dysregulation of the immune response, putting the individual in danger of multi-organ failure (MOF) and death, either because of severe inflammation or, at a later stage, due to unresolved infection [1].

Sepsis-associated immune dysregulation involving hyper-inflammation and immunosuppression is common in intensive care patients [1]. Despite significant advances in our understanding of the immune system, progress in the treatment of sepsis has been rather modest [2]. Recently, however, the achievements of targeted cancer immunotherapy have been extrapolated to the realm of sepsis with varying degrees of success [5–8]. Several immunotherapies have undergone clinical trials in sepsis with promising results, including recombinant interleukin-7 (IL-7), interferon-gamma (IFN-γ), Fms-like tyrosine kinase 3 ligand, and chimeric antigen receptor T cell (CAR-T) [5]. Notably, there is a growing appreciation of the role of immunosuppressive mechanisms in causing or exacerbating sepsis, especially that of T cell exhaustion during chronic antigenic stimulation. A therapy that modulates pathways operating in T cell exhaustion—for example, antibodies that bind to the programmed cell death 1 receptor (PD-1) on T lymphocyte surface or its ligand (PD-L1)—can reverse this dysfunctional state and reinvigorate immune responses. This understanding has led researchers to examine the potential of immune checkpoint blockers, such as PD-1/PD-L1 blockers, in treating sepsis [5, 9].

The objective of this study was to help streamline these new therapeutic approaches by identifying the main driving force underlying immunosuppression in sepsis, and by investigating possible therapeutic modalities deemed to affect this driving force. Due to the convoluted nature of the different negative and positive feedback effects involved in the key interactions in this system, analysis by naked intuition is not sufficient for disentangling the drug/disease/immunity complex. In contrast, a comprehensive mathematical model, formalizing the mechanism of action of the drug in conjunction with the pertinent host and disease processes in one succinct computational framework, would enable analysis of the yet unexplained phenomena associated with sepsis and its regulation. The major concept underlying the mathematical modeling of a complex biological system is parsimony: only the most important forces in the system are formulated, assuming that other forces have little influence on the system’s pertinent dynamics, and therefore do not add quality. Having formulated and analyzed the model, it is then validated by independent data, in order to test the assumption that the forces taken account of are the most relevant to the investigated dynamics. Only if model predictions are refuted, then the model is extended by additional assumptions. Models of this kind have proven useful for this purpose in a wide range of medical fields, by offering a broader understanding of the pathologies being studied, and by allowing to examine a diverse set of targeted interventions in the complex systems at hand [10].

Thus far, mathematical modeling in sepsis has served to explore various immunologic effects. These include the effect of the intracellular toll-like receptor 4 (TLR4) on the shift of equilibrium between the pro- and anti-inflammatory signaling cascades, the effect of cytokine perturbations on the mortality rate of the pathogen, etc., [11–13]. However, previous mathematical models developed for sepsis do not directly deal with the immunosuppression issue, and few of them use the level of simplicity suggested here for crystalizing the decisive system dynamics [14, 15].

In this work, we developed models of sepsis-associated inflammation, and used them to investigate potential drivers and inhibitors of immunosuppression. Our chosen modeling method consciously adopts a low-resolution outlook, building what one could dub a “skeletal model,” that is, a model that incorporates only the bare bones of the system. By doing so, we could more easily isolate our chosen variables and analyze the overarching dispositions of the system. Therefore, these models deliberately attempt to include the minimal number of compartments while retaining the fidelity of description, allowing us to achieve our stated goal. Simulations of these parsimonious sepsis models suggest that immunosuppression can occur only if the persistent pathogen causes superfluous T cell death, and that one can prevent sepsis-associated immunosuppression and prolonged pathogen presence by timely coupling antibiotics with immune checkpoint blockers, but not by either one of these two treatment modalities alone.

## Models and results

We aimed to develop simple mathematical models for deciphering the major forces causing immunosuppression in sepsis and improving treatment approach toward alleviating the syndrome and eliminating the pathogen. Underlying our model development was the assumption that immunosuppression in septic patients is a result of dynamic interactions between the pathogen and cells in two hematopoietic lines—myelopoiesis and lymphopoiesis [16, 17]. Our rationale was to develop a skeletal model—model V1—and to test it for retrieving immunosuppression associated with pathogenic infection. If model V1 failed to depict this desired scenario, we would then modify it and develop model V2, containing a key change to model V1. In model V2, pathogen-driven apoptosis of lymphocyte plays a major role. The difference between these two sets of results is what points to the essentiality of cell death in the etiology of immunosuppression in sepsis.

### Model V1

In this model, we assumed that the invading pathogen (P) is detected and disrupted by myeloid cells (M; neutrophils and macrophages), which die by pathogen phagocytosis [16–18]. Since the binding of pro-inflammatory cytokines—among them granulocyte colony-stimulating factor (G-CSF) and granulocyte-macrophage colony-stimulating factor (GM-CSF)—depends on myeloid abundance, significant neutrophil mortality in the peripheral blood during unremitting infection can result in increased circulation of free G-CSF to the bone marrow [19]. Ultimately, this leads to binding of pro-inflammatory cytokines to hematopoietic stem cells (HSCs, H), causing them to produce higher levels of myeloid cells at the expense of the lymphoid lineage, by shifting their differentiation ratio towards the myeloid lineage [20]. The pathogen itself also causes secretion of G-CSF and GM-CSF, by presenting on its cell surface characteristic molecules termed pathogen-associated molecular patterns (PAMPs) that are recognized by mature myeloid cells in blood, as well as by other cells in the body [21]. The myeloid cells are induced to secrete selected cytokines, such as IFN-*γ*, tumor necrosis factor-*α* (TNF-*α*), etc., which regulate activation of the adaptive immune response, finally providing protection to the host. In our model, we have compressed these processes into one—the bias caused by the pathogen in the differentiation of HSC toward the myeloid developmental line.

The pool of HSCs is kept in balance in the bone marrow by their circulation in the bloodstream to find new niches, sometimes at distant locations in the body [22]. Based on this knowledge, the model treats the size of the HSC pool as a constant parameter. We further assumed that when the innate immune system is unable to eliminate the pathogen, it activates a certain sub-population of circulating naïve T lymphocytes (L) [23]. Our model takes account of the mechanism of this activation, mediated mainly by antigen presenting cells (APCs), such as macrophages and dendritic cells in the myeloid compartment. This sub-population then undergoes clonal expansion [24, 25]. Recently, it has been reported that B cells play a more important role in sepsis than previously thought [26]. Conceptually, the effect of these cells can be readily incorporated into our lymphocyte variable, *L*, which for the sake of parsimony was not split into the various relevant lymphocyte subpopulations.

The pathogen (P) itself was taken as proliferating within the organism up to its maximum carrying capacity, that is, according to a logistic growth function (see Additional file 1 and, e.g., [12]). In addition, we assumed that myeloid cells and lymphocytes all the while continually kill the pathogen. We hypothesized, for simplicity, that the pathogen can decrease to exceedingly low levels but cannot be completely eliminated, so that it can recover when the immune system is suppressed. Note, however, that our “pathogen” variable does not necessarily represent the same pathogen throughout the entire time-period simulated. Rather, this variable can stand for re-emergence of the previous pathogen, e.g., from organs or tissues where antibiotics penetrate poorly, or for a new pathogen hitherto not encountered by the patient’s immune system.

**Fig. 1:**
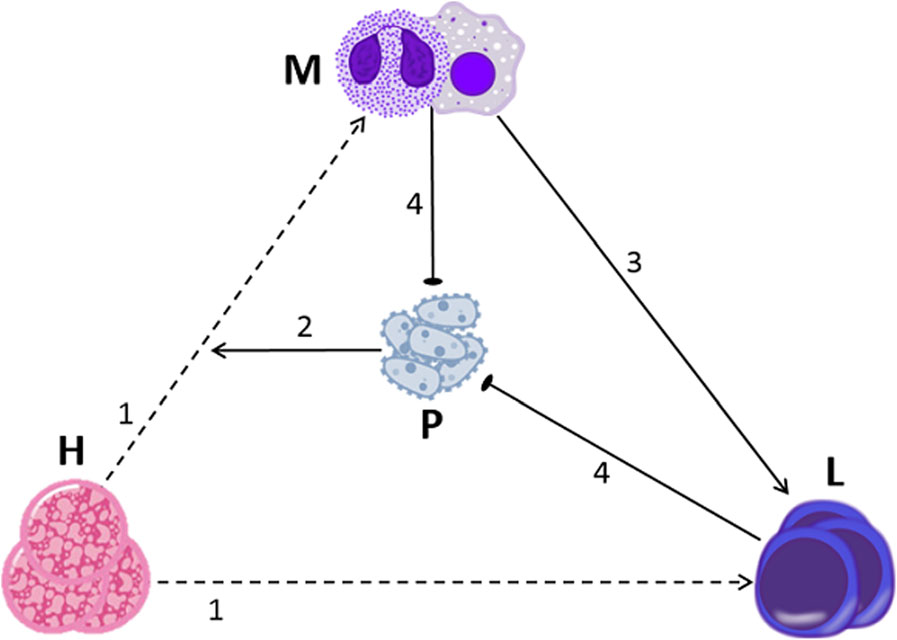
A graphical display of Model V1. The model is based on four assumptions: 1. HSCs (H) continually differentiate into myeloid cells (M; neutrophils, macrophages) and lymphocytes (L). 2. The presence of pathogen (P) biases HSC differentiation towards the myeloid lineage. 3. Lymphocytes encounter myeloid cells that have phagocytized antigen, and expand their population in response. 4. Myeloid cells and lymphocytes inhibit pathogen growth. See Additional file 1 for the equations and parameters for Model V1. Regular, blunted, and dashed arrows indicate activation, inhibition, and differentiation, respectively

We formed a model, succinctly describing all the effects noted above (model V1); the model is depicted in Fig. 1.

Following the above diagram, we designed a set of differential equations, which formulate the processes described in the model (Table 1).

**Table 1:** Equations for model V1. Parameter definitions and values: HSC population, *H* = 0.5; homeostasis probability of HSC differentiation into a myeloid cell, *a*_*M*_ = 0.2; myeloid death rate, *μ*_*M*_ = 0.025; lymphocyte proliferation rate, *p*_*L*_ =0.2; lymphocyte death rate, *μ*_*L*_ = 0. 4; pathogen proliferation rate, *p*_*P*_ values vary. Pathogen killing rate by myeloid cells, *κ*_*M*_ = 0.6; pathogen killing rate by lymphocytes, *κ*_*L*_ = 1; HSC skew regulator, *α* = 0.8; rate of APC stimulation of lymphocytes, *β* = 0.2. See Additional file 1 for a concise summary and explanation of the equations and parameters for all models presented in this article

Variable/function	Equation	Initial value
Myeloid cells (*M*)	$\frac{dM}{dt}={f}_1(P)\cdotp {a}_MH-{\mu}_MM$	*M*(*t* = *o*) = 4
Lymphocytes (*L*)	$\frac{dL}{dt}=\left(1-{f}_1\cdotp {a}_M\right)H+{f}_2(M)\cdotp {p}_LL-{\mu}_LL$	*L*(*t* = *o*) = 2
Pathogen (*P*)	$\frac{dP}{dt}={p}_PP\left(1-\frac{1}{P_{\infty }}\right)-{\kappa}_MM\frac{P}{k+P}-{\kappa}_LL\frac{P}{k+P}$	*P*(*t* = *o*) = 3
HSC differentiation skew	${f}_1(P)=\frac{1+\frac{\alpha }{a_M}P}{1+P}$	*f*_1_(*t* = *o*) = 1
APC stimulation of lymphocytes	*f*_2_(*M*) = 1 + *β*(*M* − *M*_0_)	*f*_2_(*t* = *o*) = 1

**Fig. 2:**
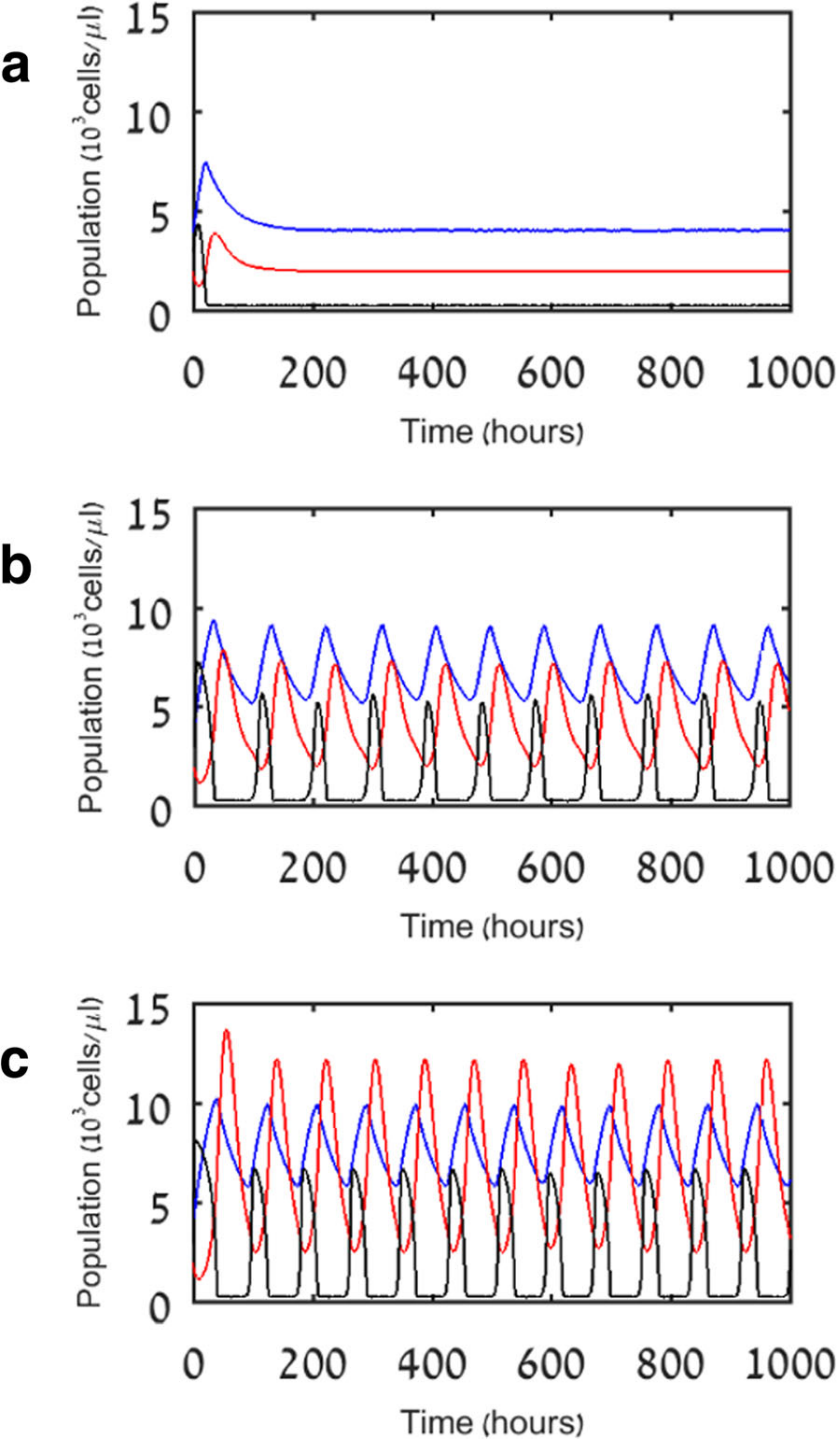
Simulation results of model V1 for pathogens differing in growth rate. Results are shown for three cases of pathogen growth rate, p_P_: **a** mild pathogens (p_P_ = 1); **b** moderate pathogens (p_P_ = 1.5); **c** aggressive pathogens (p_P_ = 2). Blue, red, and black lines indicate myeloid cells (M), lymphocytes (L), and pathogen (P), respectively

We divided pathogens into three categories, which vary in their proliferation rate (labeled p_P_): mild (p_P_ = 1), moderate (p_P_ = 1.5), and aggressive (p_P_ = 2). Figure 2 shows results for model V1, illustrating dynamics of the system over six weeks for the three main populations of interest: pathogen, lymphocyte, and myeloid cells. We found that the immune system is able to permanently eliminate mild pathogens, thereafter approaching homeostasis levels (Fig. 2a), while moderate (Fig. 2b) and aggressive pathogens (Fig. 2c) manage to recover once the immune cells return to their homeostatic levels, stimulating another immune response. The latter two cases approach a limit cycle, namely oscillations of the three cell populations, having the same phase and almost a constant amplitude, with no change in the average abundance of the pathogen or the blood cells. We ran all simulations using MATLAB R2016a. All cell populations are in units of 10^3^ cells/μl. All time units are hours.

Clearly, then, model V1 does not account for persistent pathogenesis accompanied by immunosuppression; the latter is expected to manifest as lymphocytes’ gradual diminution. Although the lymphocyte compartment suffers from low supply from the bone marrow relative to normal (see above), this is compensated for by the enhanced activation of naive T cells by APCs in the myeloid compartment. We therefore examined possible alterations to this model, to reflect the phenomenon observable in patients.

### Model V2

**Fig. 3:**
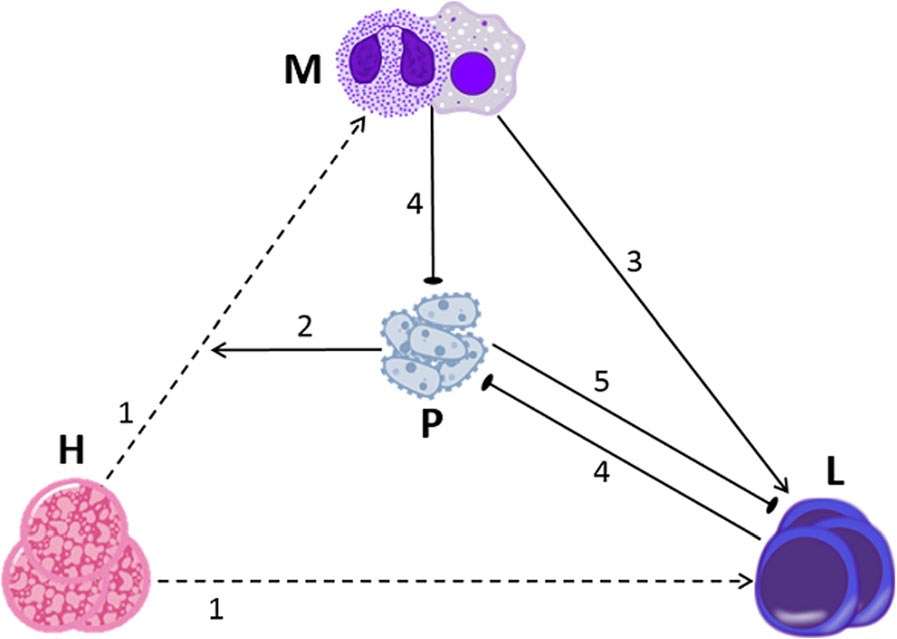
A graphical display of Model V2. The only change from model V1 (see Fig. 1) is the addition of effect 5, by which persistent pathogen induces lymphocyte exhaustion, via suppression of HLA-DR expression in APCs or through the PD-1/PD-L1 pathway. Effectively, exhaustion in the model manifests itself as an increased mortality rate of lymphocytes. See Additional file 1 for the equations and parameters of model V2. Regular arrows indicate activation; blunted arrows indicate inhibition; dashed arrows indicated differentiation

Having examined various modeling options and their associated system’s dynamics, we finally chose to incorporate one more effect in the above-described skeletal model: T cell exhaustion. Thus, in the new model (model V2), we added the assumption that during prolonged infection, persistent pathogen presence induces T cell exhaustion, which over time inhibits the expansion of lymphocytes [7]. Figure 3 illustrates our more complex model V2, with arrows 1–4 identical to those in model V1 and arrow 5 representing the assumption of T cell exhaustion by the pathogen.

It was now also necessary to modify our equations to fit the new model. Table 2 contains the equations formulated for model V2. Note that the only change made to the initial equations is the addition of the exhaustion function and its incorporation in the lymphocyte equations.

**Table 2:** Equations for model V2, which differs from model V1 in having an additional assumption of lymphocyte exhaustion process. Parameter definitions and values: HSC population, *H* = 0.5; homeostasis probability of HSC differentiation into a myeloid cell, *a*_*M*_ = 0.2; myeloid death rate, *μ*_*M*_ = 0.025; lymphocyte proliferation rate, *p*_*L*_ =0.2; lymphocyte death rate, *μ*_*L*_ = 0. 4; pathogen proliferation rate, *p*_*P*_ values vary. Pathogen killing rate by myeloid cells, *κ*_*M*_ = 0.6; pathogen killing rate by lymphocytes, *κ*_*L*_ = 1; HSC skew regulator, *α* = 0.8; rate of APC stimulation of lymphocytes, *β* = 0.2. Exhaustion effect increases in presence of pathogen. Rate of increase in exhaustion effect, *γ*_1_ = 0.005; degree to which pathogen affects onset of exhaustion, *γ*_2_ = 100; rate of dissipation of exhaustion effect, *μ*_*exh*_ = 0.002. See Additional file 1 for a concise summary and explanation of the equations and parameters for all models presented in this article

Variable/function	Equation	Initial value
Myeloid cells (*M*)	$\frac{dM}{dt}={f}_1(P)\cdotp {a}_MH-{\mu}_MM$	*M*(*t* = *o*) = 4
Lymphocytes (*L*)	$\frac{dL}{dt}=\left(1-{f}_1\cdotp {a}_M\right)H+{f}_2(M)\cdotp {p}_LL-\left(1+ exh\right)\cdotp {\mu}_LL$	*L*(*t* = *o*) = 2
Pathogen (*P*)	$\frac{dP}{dt}={p}_PP\left(1-\frac{1}{P_{\infty }}\right)-{\kappa}_MM\frac{P}{k+P}-{\kappa}_LL\frac{P}{k+P}$	*P*(*t* = *o*) = 3
HSC differentiation skew	${f}_1(P)=\frac{1+\frac{\alpha }{a_M}P}{1+P}$	*f*_1_(*t* = *o*) = 1
APC stimulation of lymphocytes	*f*_2_(*M*) = 1 + *β*(*M* − *M*_0_)	*f*_2_(*t* = *o*) = 1
Exhaustion function dynamics	$\frac{dexh}{dt}=\frac{\gamma_1}{1+{e}^{-{\gamma}_2\left(P-1\right)}}-{\mu}_{exh} exh$	*exh*(*t* = 0) = 0

**Fig. 4:**
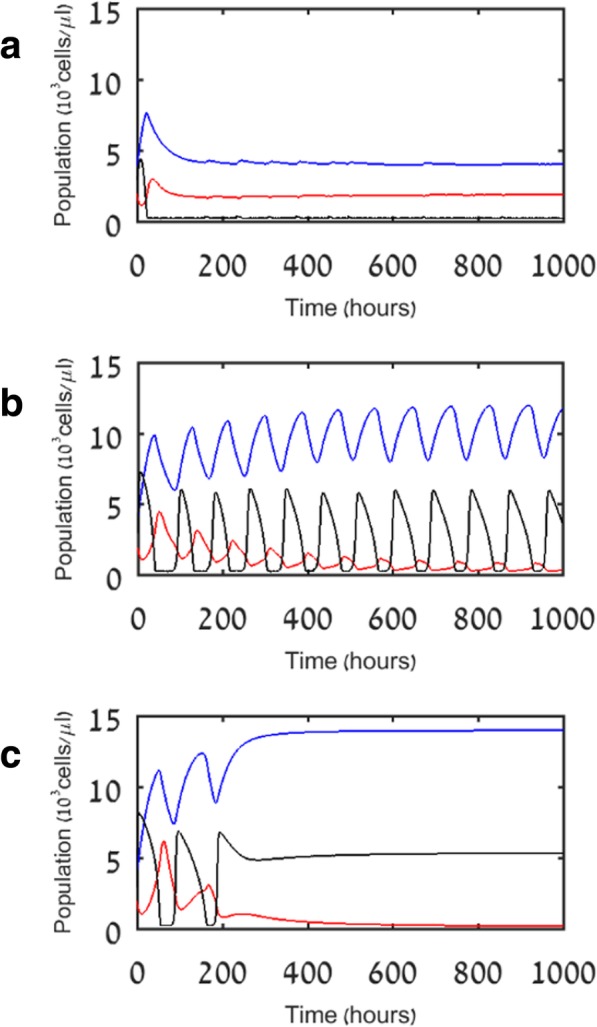
Simulation results for model V2. **a** Mild pathogens (p_P_ = 1) are quickly eliminated. **b** Moderate pathogens (p_P_ = 1.5) induce regular oscillations of the pathogen with a slowly declining lymphocyte population. **c** Aggressive pathogens (p_P_ = 2) cause a rapid lymphocyte decline, allowing the pathogen to remain at a constant high level (as opposed to the result in Fig. 2c). Blue, red, and black lines indicate myeloid cells (M), lymphocytes (L), and pathogen (P), respectively

Figure 4 shows simulation results of model V2. As in model V1, here in addition, the immune system is able to extinguish mild pathogens, while moderate and aggressive pathogens manage to recover, generating another immune stimulation and a new suppression of the pathogen. However, in contrast to the results of model V1, we note that when the pathogen is moderate or aggressive, the simulations of model V2 show a progressively weakening lymphocyte population. In fact, for aggressive pathogens, the system quickly abandons the limit cycle and approaches stability of myeloid cell numbers simultaneously with progressive lymphocyte decay, in keeping with the paradigm that hyper-inflammation and immunosuppression can occur simultaneously [5].

Interestingly, Fig. 4 shows that under moderate pathogen proliferation rates, the pathogen oscillates almost steadily between low and high abundance. Under larger pathogen proliferation rates, its abundance stays high and slowly increases. Note that the value of *P* at *t* = 0, i.e., the initial level of the infection, is a less influential factor than the pathogen proliferation rate (p_P_). To illustrate this, we simulated the model as in Fig. 4 above, except that the initial pathogen population size was increased to *P* = 10 at *t* = 0 (rather than *P* = 3 in Fig. 4). Simulation results with initial pathogen at *P* = 10 are qualitatively almost identical to those from Fig. 4, demonstrating that the virulence of the pathogen is more important than the initial pathogen load (not shown; see Additional file 2).

**Fig. 5:**
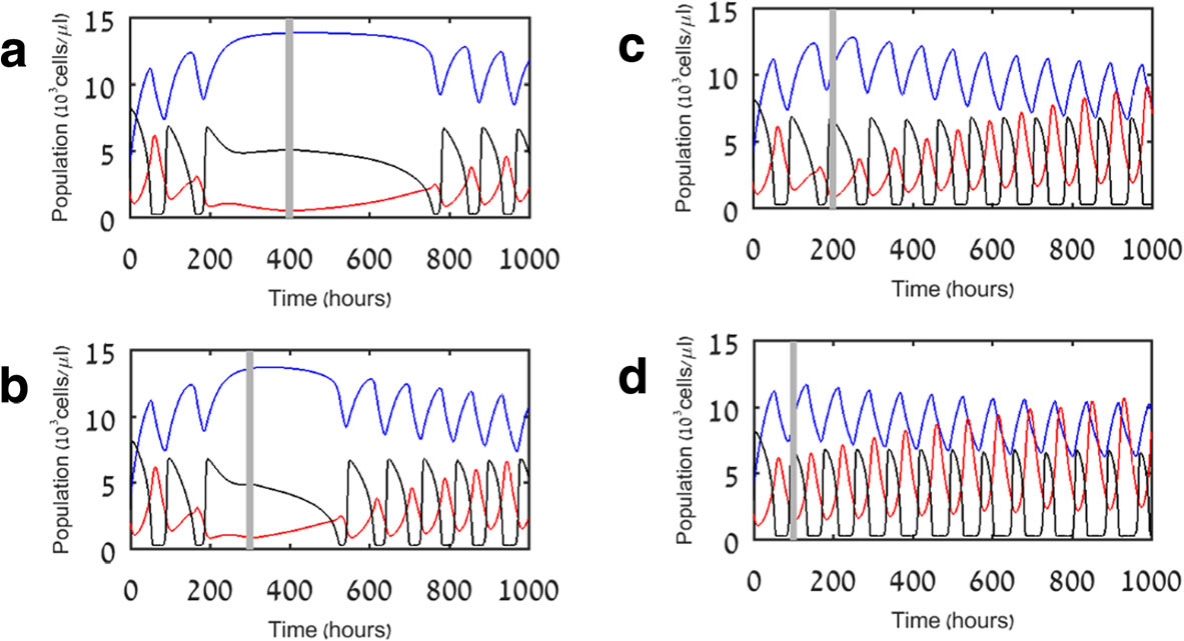
Simulation results of model V2 for treatment with PD-1/PD-L1 checkpoint blockers at different time points during sepsis. Blue, red, and black lines indicate myeloid cells (M), lymphocytes (L), and pathogen (P), respectively. Vertical straight lines indicate the time at which immunotherapy is administered: **a** 400 h; **b** 300 h; **c** 200 h; **d** 100 h

In the next stage, we used model V2 for studying the effect of different treatment modalities on eliminating the pathogen, and on alleviating the immunosuppression. In Fig. 5, we show simulation results of model V2 for the effects of PD-1/PD-L1 checkpoint blockers on aggressive pathogens. We assumed that immunotherapy by PD1/PD-L1 blockers has the effect of halting the process of T cell exhaustion and enabling lymphocyte reinvigoration. This assumption was formalized in the equations as an immediate elimination of the positive element in our exhaustion function, *exh*, so it became $\frac{dexh}{dt}=-{\mu}_{exh} exh$ (see Table 2), allowing the remaining negative linear element to slowly reduce it to zero. Observe that this is equivalent to a gradual reversion back to the equations of model V1, which as stated above does not include lymphocyte exhaustion.

Moreover, for simplicity, we assumed that once the immunotherapeutic drug is applied, its effect persists throughout the 6 weeks’ follow-up period (see Additional file 1 for details of the mathematical model formulation of this effect).

First, we wished to check whether the timing of immunotherapy treatment could determine the fate of the system. To do so, we applied a similar immunotherapy in different time points during sepsis of the same “patient.” Figure 5 suggests that treatment by checkpoint blockers alone can relieve the immunosuppression and reduce the myeloid overflow, but cannot annihilate the most aggressive pathogens simulated in this example. The results in Fig. 5 underline a therapeutic window in which the immunotherapy can be most efficacious. When treatment is administered as late as 400 h after diagnosis of sepsis, there is some late improvement and the immune system eventually recovers, but the patient is exposed to continuous infection and hyper-inflammation by myeloid cells for an unreasonably long period and therefore the recovery is too slow to be clinically meaningful. The dynamics of the immune cells and the pathogen are different upon an earlier immunotherapy application. Treatment at 300 h causes pathogen levels to fall more sharply, patients return to an oscillatory state within reasonable time, but are not cured. Treatment at 200 h is sufficient to prevent pathogens from consistently sustaining a high level at all times and instead maintains the oscillatory state with lymphocyte numbers slowly recovering, while treatment at 100 h enables to prevent the initial drop in lymphocytes and the overshoot in myeloid cells.

**Fig. 6:**
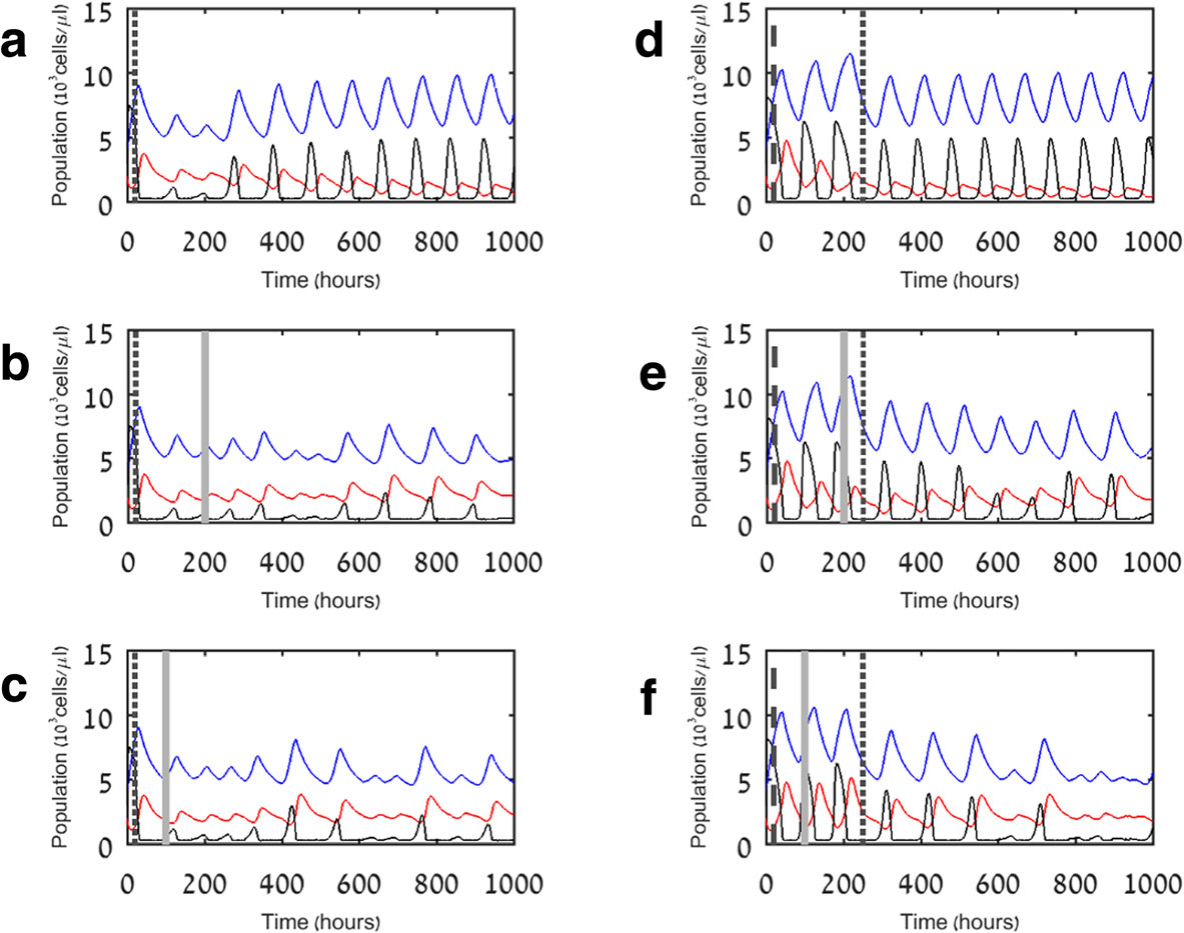
Simulation results for treatment with different protocols of antibiotics and their combination with immunotherapy. **a** Targeted antibiotics alone. **b** Targeted antibiotics followed by immunotherapy after 200 h. **c** Targeted antibiotics followed by immunotherapy after 100 h. **d** Targeted antibiotics followed by targeted antibiotics after 250 h. **e** Targeted antibiotics followed by immunotherapy after 200 h and targeted antibiotics after 250 h. **f** Targeted antibiotics followed by immunotherapy after 100 h and targeted antibiotics after 250 h. Blue, red, and black lines indicate myeloid cells (M), lymphocytes (L), and pathogen (P), respectively. Dotted vertical lines indicate time of administration of strong targeted antibiotics (reduction of 40% in pathogen growth rate, p_P,_ to 1.2); straight vertical gray lines indicate time of administration of immunotherapy; dashed vertical lines indicate time of administration of weak broad spectrum antibiotics (reduction of 20% in pathogen growth rate, p_P_***,*** to 1.6)

Above we noted that under immunotherapy alone, the aggressive pathogen would never be fully defeated, since it is capable of recovery once immune cells naturally return to their initial levels. Remembering that antibiotics are the first line drugs for sepsis, we also examined the effect of several different protocols of antibiotics applied as monotherapy, or in combination with immunotherapy [27]. The first protocol consists of efficacious antibiotics, i.e., directed to the patient’s specific pathogen, administered almost as soon as the initial infection is detected (20 h). The second protocol simulates a more realistic situation, where several days are needed for an accurate diagnosis of the pathogen, and so the antibiotics administered at first (20 h) are of a less efficacious, broad-spectrum type. However, at 250 h, a targeted antibiotic drug is administered, replacing the weaker drug. The term “targeted” is used for pathogen-specific antibiotics, that is, ones that were developed to target a certain pathogen rather than to be effective against a wide variety; targeted antibiotic treatment is usually considered to be more efficacious. The reason these drugs are administered later in our “realistic scenario” simulations (Fig. 6d–f) is that time must be allowed for diagnosis. In these simulations, we also assumed for simplicity that once an antibiotic drug is applied, its effect remains steady throughout the patient’s stay in the intensive care unit (ICU; ca. 6 weeks). This simulation also represents multiple applications of the same antibiotic drug. The formulation of the antibiotics’ effect is a reduction in the pathogen’s proliferation rate, *p*_*P*_ (see Table 2) by 20% for the weaker drug and 40% for the stronger drugs.

Figure 6a examines the potential of an early dosing of strong antibiotics to an aggressive pathogen. We see that they temporarily reduce the pathogen to almost elimination but the decline in lymphocytes allows the pathogen to recover and achieve oscillations at an amplitude that could be harmful to the patient. This is still qualitatively a better result than no treatment, where the pathogen oscillates with non-decaying amplitudes before converging upon a permanent high level (compare to Fig. 4c). How will a combination with of immunotherapy affect the prognosis? Figure 6b, c shows results for treatment of strong antibiotics, combined with a PD1/PD-L1 blocker applied at two alternative time intervals. These results indicate a significant reduction of the pathogen with occasional small spikes. In addition, the rise in myeloid cells is kept at bay and there is no visible influence of the timing of immunotherapy application. Figure 6d–f shows results for the more realistic antibiotics protocol we devised (see above), which includes weak antibiotics at the early stages but delays the strong antibiotics. We see in Fig. 6d that without immunotherapy, the result is oscillations, similar to those in Fig. 6a—the pathogen is somewhat weakened but is not eliminated. Adding immunotherapy following the nonspecific antibiotics (Fig. 6e, f) significantly hampers the pathogen’s recovery and, following application of the strong antibiotics, we see that with early onset of immunotherapy, this treatment is no less efficacious than our optimistic scenario of early strong antibiotics with early immunotherapy (compare Fig. 6f to c). This demonstrates how the immunotherapy, by reinvigorating the exhausted lymphocytes, can significantly contribute to the treatment. With administration of immunotherapy sufficiently early during the waiting time until specific antibiotic drug is identified, T lymphocytes increase in numbers to become more effective in eliminating the invading pathogen. The more specific antibiotic drug meets a less abundant pathogen and a more vigorous cellular immunity. Therefore, its effect, combined with that of the immunotherapy, can eliminate the highly proliferative pathogen despite the delay in its application. One can view this approach as “buying the patient time” to allow a partial recovery, while being more accurately diagnosed. Note also that under the second, more realistic antibiotics protocol, results are significantly better with checkpoint blockers administered at 100 h than at 200 h. This illustrates the importance of timely onset in immunotherapy.

## Discussion and conclusions

According to our results, the key point of vulnerability of the immune system when facing aggressive pathogens is the potential for lymphocyte exhaustion [28]. This is the important result of our parsimonious modelling. Without the assumption that the persistent pathogen presence induces T cell exhaustion, the simulation results with realistic parameter values failed to reflect cases in which immunosuppression occurs (model V1). In other words, T cell exhaustion caused by the pathogen is the simplest known mechanism (e.g., [29]), which can drive sepsis-mediated immunosuppression. One can also conclude that myeloid cells alone are insufficient for alleviating severe sepsis and reasonable levels of lymphocytes are essential for containing the pathogen. In model V2, ongoing pathogen presence mediates lymphocyte exhaustion. It should however be pointed out that other conditions exist which can play a similar role. In particular, conditions such as acute pancreatitis or post-surgical trauma, via the resultant release of damage-associated molecular patterns, are liable to induce lymphopenia [30–32]. Due to our choice of variables and our desire to keep our model “skeletal,” these types of effects were outside the scope of our consideration.

The results also illustrate the plausibility of concurrent hyper-inflammation and immunosuppression, which manifest simultaneously in the myeloid and lymphoid compartments. In the event of severe infection, these two syndromes could create a positive feedback loop, where immunosuppression prevents pathogen elimination, thereby further boosting inflammatory activity [1]. Prolonged inflammation may ultimately lead to catastrophic events of MOF [2].

Our results further show that the immune system is able to eliminate mild pathogens permanently, while moderate and aggressive pathogens can recover and drive the system to oscillatory dynamics of the three cell populations. We believe that this result merits clinical validation as it can have therapeutic implications.

When exploring the therapeutic option of anti-exhaustion treatment by PD-1/PD-L1 blockers, we found that with aggressive pathogens, early intervention—which arrests the exhaustion effect—changes the projection for pathogen from constant high levels to periodic oscillations between escalation and decline. This could reduce the potential damage to the patient, both from the pathogen itself and from the ensuing inflammation. The second monotherapy treatment examined, antibiotics, also proved capable of reducing pathogen levels from constant to oscillatory. From a biological perspective, the pathogen’s ability to recover under these conditions, can be attributed to a number of factors: bacteria may survive inside organs or tissues where antibiotics penetrate poorly, resistant strains may proliferate, or new opportunistic organisms may cause secondary infection during the periods when the patient’s immune state is compromised [33].

Combination of antibiotics with checkpoint inhibitors, if administered early enough, virtually eliminates the pathogen in our model system. In particular, the early administration of the immunotherapy is essential for significantly accelerating the recovery of lymphocytes. These results appear to align with contemporary observations and clinical reality [27, 34]. Our results show that application of a PD-1 blocker at the earliest clinically permissible time point is the only schedule, out of the four tried, to prevent the initial drop in lymphocytes and the overshoot in myeloid cells. The reasoning is the “race of arms” between cellular immunity and the pathogen—the earlier one applies immunotherapy, the fewer lymphocytes have already been annihilated by the pathogen and the larger is the potential reinvigoration the applied checkpoint inhibitor can cause to living but already partly exhausted lymphocytes. However, the clinical limitations of check-point inhibitors should be noted, in particular the possibility that these drugs may “overshoot” and induce potentially fatal autoimmune reactions [35].

Another motivation we had in developing the present model was furthering the understanding of immunosuppression in sepsis. We made a particular effort to build the narrowest possible model that could capture this phenomenon. As demonstrated above, this approach allowed us to identify the key forces in the system—leading us to the discovery that lymphocyte exhaustion due to pathogen persistence is the crucial effect required for the model to reflect the pathologies we were studying. However, the “skeletal model” developed in this work can provide the groundwork for developing more complex models which might then lend themselves to be personalized. Options for broadening the model include expanding the hematopoietic differentiation process by adding intermediary progenitors between HSCs and mature leukocytes (see, e.g., [36, 37]); considering the separate effects of myeloid derived suppressor cells (MDSCs) and apoptotic cells [38, 39]; including other types of cells (such as dendritic cells). Note, however, that while such improvements may enable a better liaison of personally measured parameters with the mathematical model, we do not expect them to alter the conclusions drawn in this work. Presently, we examined a few types of monotherapy and combined treatments, but the model is flexible and may be adapted to shed light on other therapeutic possibilities as well (see [40, 41] for examples).

Further potential projects that build on our model also include optimizing the timing of treatments and fitting the model parameters to patients’ clinical data. Our limited analysis brought forward here provides some indication as to parameter sensitivity when personalizing the model to individual patients; a priori, reaction rates that are pertinent to lymphocyte exhaustion seem to be most sensitive to the propensity to suffer immunosuppression. As well as these possibilities, one could also use our model to guide the design of clinical trials (e.g., [42]).

We will validate our model’s predictions in several retrospective and prospective preclinical and clinical trials. First among them is a retrospective clinical trial, validating the numerical accuracy of model-predicted longitudinal blood counts. We also intend to use this trial for tuning the model’s parameters more finely. The trial is currently in process, involving a large database of ICU patients (MIMIC-III, a freely accessible critical care database [43]). Next, we will carry out prospective preclinical and clinical trials to test the qualitative validity of model predictions. In the preclinical setting, selection of an in vivo small animal model of human sepsis and human immune system interaction would enable verification of the mechanisms underlying immunosuppression in sepsis. In addition, we will examine in the preclinical setting the treatment protocol our study highlights as optimal for severe sepsis. However, the predictive validity and translational value of animal models for the medical research in sepsis is a subject of considerable debate, at present: it is essential to acknowledge that all experimental models have limitations and that an animal model can never fully replicate all of the features of human disease [44]. Therefore, our aim will be to carry out prospective clinical trials for validating the superiority of the model-suggested improved regimen, combining immunotherapy and antibiotics.

## Conclusions

The results from our model simulations suggest that the key cause of immunosuppression in septic patients is lymphocyte exhaustion, and that an early onset of antibiotic treatment sequenced with an early treatment for blocking T cell exhaustion, such as PD-1/PD-L1 checkpoint blockers, can concomitantly alleviate this undesirable effect and sepsis. In particular, anti-exhaustion treatment can provide the key ingredient for disease resolution when antibiotics alone are insufficient or when it takes time to find the best antibiotics to the patient’s specific disease. Following preclinical and clinical validation, our model can be adapted to explore the potential of other therapeutic options in this field.

## Additional files

Additional file 1: A concise summary and explanation of the equations and parameters for all models presented in this article. (DOCX 13 kb)

Additional file 2: **Figure S1.** Simulation results for Model V2 referred to under Fig. 4 in main article. a) Mild pathogens (p_P_ = 1) b) moderate pathogens (p_P_ = 1.5) c) aggressive pathogens (p_P_ = 2). Blue indicates myeloid cells (M); red indicates lymphocytes (L); black indicates pathogen (P). Initial conditions: M=4; L=2; P=10. The last value is in contrast to the simulation shown in Fig. 4 where the initial value for P is 3. (TIF 180 kb)

## Abbreviations

p_P_: Pathogen proliferation rate
APC: Antigen presenting cell
CAR-T: Chimeric antigen receptor T-cell
G-CSF: Granulocyte-colony stimulating factor
GM-CSF: Granulocyte macrophage-colony stimulating factor
HSC or H: Hematopoietic stem cell
ICU: Intensive care unit
IFN-*γ*: Interferon-gamma
IL-7: Interleukin-7
L: Lymphocytes
M: Myeloid cells
MDSC: Myeloid derived suppressor cells
MOF: Multiple organ failure
P: Pathogen
PAMP: Pathogen-associated molecular pattern
TLR4: Toll-like receptor 4
TNF-*α*: Tumor necrosis factor-alpha
