# Supplementary material for: Alleviation of exhaustion-induced immunosuppression and sepsis by immune checkpoint blockers sequentially administered with antibiotics—analysis of a new mathematical model
Source: Intensive Care Med Exp. 2019 Jun 11;7:32. doi: 10.1186/s40635-019-0260-3 (PMC6560115; doi:10.1186/s40635-019-0260-3)
Supplement: Supplementary file 1 — A concise summary and explanation of the equations and parameters for all models presented in this article. (DOCX 13 kb) [file 40635_2019_260_MOESM1_ESM.docx]

Description of models and mathematical equations

**Model V1.**

Change in myeloid cell blood population ($\dot{M}$) is affected by two elements: the rate of supply from the hematopoietic stem cell (HSC) compartment ($H$), and the myeloid death rate ($\mu_{M}$), which is proportionally linear to the mature cell population. The monotone function $f_{1}$, which depends on pathogen ($P$), dictates the HSC supply. Thus when $P=0$ the probability of each differentiating HSC maturing into a myeloid cell is the homeostasis value $a_{M}$, while when $P\to\infty$ this probability approaches $\alpha$.

Change in lymphocyte blood population ($\dot{L}$) has two positive elements and one negative. As with myeloid cells, the negative element is a linear death rate ($\mu_{L}$) while the first positive element is a supply from the HSC compartment, with the differentiation probability coefficient being the complement to its myeloid counterpart. In addition there is a proliferation element, which is linear in $L$ but also affected by myeloid cells through the function $f_{2}$ (via antigen representation).

Change in pathogen blood levels ($\dot{P}$) is affected positively by a logistic growth function of the pathogen, and negatively by two similar elements that represent the rate at which pathogen is killed by myeloid cells and lymphocytes. These elements are proportional to their respective immune cell populations and to a hyperbolic function of the pathogen.

Myeloid cell equation: $\dot{M}={f_{1}\left( P \right)\cdot a}_{M}H-\mu_{M}M$ $M\left( t=o \right)=4$

Lymphocyte dynamics equation:$\dot{L}=\left( 1-f_{1}\cdot a_{M} \right)H+f_{2}\left( M \right)\cdot p_{L}L-\mu_{L}L$ $L\left( t=0 \right)=2$

Pathogen dynamics equation: $\dot{P}=p_{P}P\left( 1-\frac{1}{P_{\infty}} \right)-\kappa_{M}M\frac{P}{k+P}-\kappa_{L}L\frac{P}{k+P}$ $P\left( t=o \right)=3$

HSC differentiation skew function: $f_{1}\left( P \right)=\frac{1+\frac{\alpha}{a_{M}}P}{1+P}$

Antigen representation function: $f_{2}\left( M \right)=1+\beta(M-M_{0})$

**Model V2.**

All equations are identical to Model V1 except the lymphocyte dynamics, where the death rate is now affected by a dynamic element representing exhaustion ($exh$). $exh$ represents the momentary effect of exhaustion on lymphocyte mortality or loss of cytotoxic function. It rises at a constant rate when $P\gg1$ (this is formulated using a sigmoid function) and dissipates at a linear rate at all times. Initial values of cell populations remain the same as in Model V1.

Lymphocyte dynamics equation:$\dot{L}=\left( 1-f_{1}\cdot a_{M} \right)H+f_{2}\left( M \right)\cdot p_{L}L-\left( 1+exh \right)\cdot\mu_{L}L$

Exhaution function: $\dot{exh}=\frac{\gamma_{1}}{1+e^{-\gamma_{2}(P-1)}}-\mu_{exh}exh$ $exh\left( t=0 \right)=0$

| Parameter | Description | Value | References |
| --- | --- | --- | --- |
| $H$ | HSC population | 0.5 | Adjusted |
| $a_{M}$ | Probability of HSC differentiation into myeloid cell | 0.2 | Adjusted |
| $\mu_{M}$ | Myeloid death rate | 0.025 | Adjusted |
| $p_{L}$ | Lymphocyte proliferation rate | 0.2 | Adjusted |
| $\mu_{L}$ | Lymphocyte death rate | 0.4 | Adjusted |
| $M_{0}$ | Level of myeloid cells in homeostasis | 4 | [1] |
| $p_{P}$ | Pathogen growth rate | 1/1.5/2 | doi: [10.1099/00221287-148-9-2705](https://dx.doi.org/10.1099/00221287-148-9-2705), <https://doi.org/10.1016/j.cub.2010.04.045> |
| $P_{\infty}$ | Maximum pathogen load | 10 | Adjusted |
| $\kappa_{M}$ | Pathogen killing rate by myeloid cells | 0.6 | Adjusted |
| $\kappa_{L}$ | Pathogen killing rate by lymphocytes | 1 | Adjusted |
| $k$ | Regulation of pathogen elimination | 4 | Adjusted |
| $exh$ | Level of exhaustion effect | Dynamic | Adjusted |
| $\mu_{exh}$ | Rate of dissipation of exhaustion effect | 0.002 | Adjusted |
| $\alpha$ | Maximum skew of HSC differentiation into myeloid lineage | 0.8 | Adjusted |
| $\beta$ | Rate of antigen presentation by APCs to lymphocytes | 0.2 | Adjusted |
| $\gamma_{1}$ | Rate of increase in exhaustion effect, asymptotic in P (i.e. when $P=\infty$) | 0.005 | Adjusted |
| $\gamma_{2}$ | Rate at which increase in pathogen regulates exhaustion | 100 | Adjusted |

Table 3. Parameter descriptions and values. Level of exhaustion effect, *exh*, represents the momentary effect of exhaustion on lymphocyte mortality. 'Adjusted' means the parameter was tuned (within a reasonable range) to give sensible results from a clinical perspective.

Treatment

**Immunotherapy.** Starting at time of administration the exhaustion dynamics equation becomes $\dot{exh}=-\mu_{exh}exh$, meaning it now only decreases with time at a linear rate, regardless of pathogen levels.

**Weak antibiotics.** Pathogen growth rate ($p_{P}$) is reduced by %20 starting at time of administration for the remainder of the simulated timespan.

**Strong antibiotics.** Pathogen growth rate ($p_{P}$) is reduced by %40 starting at time of administration for the remainder of the simulated timespan.

All cell populations are in units of 10^3^ cells / μl. All time units are hours.

All simulations were run using MATLAB R2016a.

**References**

1. CDC (2013) Labroatory Procedure Manual. https://www.cdc.gov/nchs/data/nhanes/nhanes_11_12/cbc_g_met_he.pdf. Accessed 18 Oct 2018.
