# Supplementary figures and images for: Alleviation of exhaustion-induced immunosuppression and sepsis by immune checkpoint blockers sequentially administered with antibiotics—analysis of a new mathematical model
Source: Intensive Care Med Exp. 2019 Jun 11;7:32. doi: 10.1186/s40635-019-0260-3 (PMC6560115; doi:10.1186/s40635-019-0260-3)

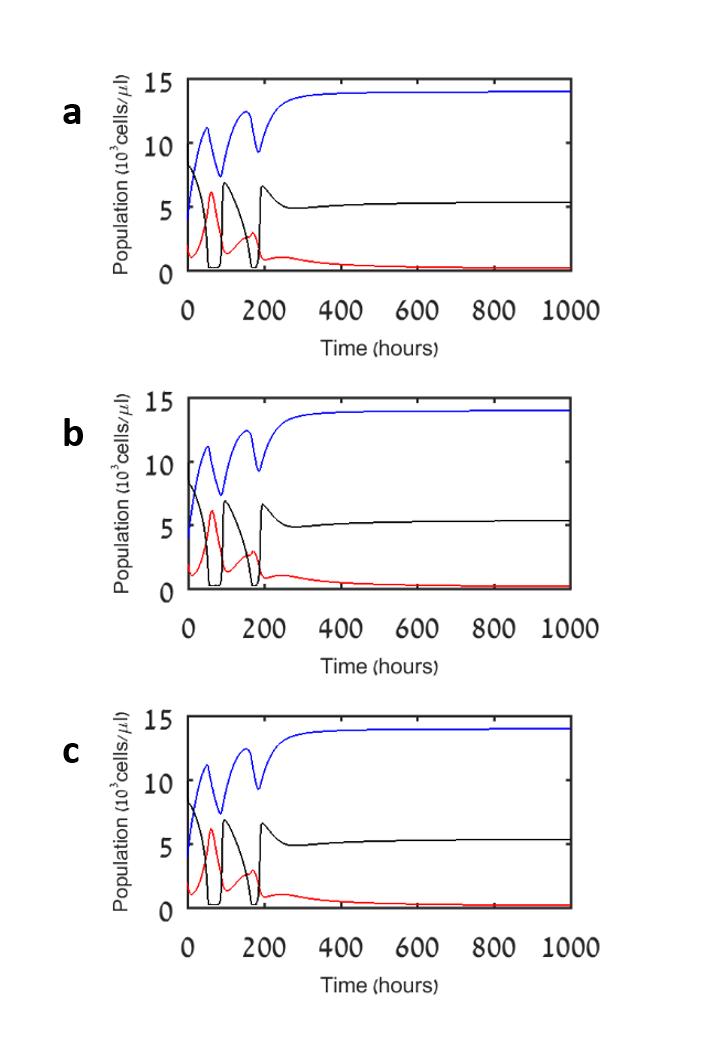

Supplement: Supplementary file 2 — Figure S1. Simulation results for Model V2 referred to under Fig. 4 in main article. a) Mild pathogens (pP = 1) b) moderate pathogens (pP = 1.5) c) aggressive pathogens (pP = 2). Blue indicates myeloid cells (M); red indicates lymphocytes (L); black indicates pathogen (P). Initial conditions: M=4; L=2; P=10. The last value is in contrast to the simulation shown in Fig. 4 where the initial value for P is 3. (TIF 180 kb) [file 40635_2019_260_MOESM2_ESM.tif]
